# Impact of the Home Confinement Related to COVID-19 on the Device-Assessed Physical Activity and Sedentary Patterns of Spanish Older Adults

**DOI:** 10.1155/2021/5528866

**Published:** 2021-05-31

**Authors:** Ángel I. Fernández-García, Jorge Marin-Puyalto, Alba Gómez-Cabello, Ángel Matute-Llorente, Jorge Subías-Perié, Jorge Pérez-Gómez, Gabriel Lozano-Berges, Asier Mañas, Amelia Guadalupe-Grau, Marcela González-Gross, Ignacio Ara, José A. Casajús, Germán Vicente-Rodríguez

**Affiliations:** ^1^GENUD (Growth, Exercise, Nutrition and Development) Research Group, University of Zaragoza, Zaragoza, Spain; ^2^Faculty of Health and Sport Sciences (FCSD, Ronda Misericordia, 5, 22001-Huesca, Spain), Department of Physiatry and Nursing, University of Zaragoza, Spain; ^3^Red española de Investigación en Ejercicio Físico y Salud en Poblaciones Especiales (EXERNET), Spain; ^4^Centro Universitario de la Defensa, Zaragoza, Zaragoza, Spain; ^5^Instituto Agroalimentario de Aragón-IA2-(CITA-Universidad de Zaragoza), Spain; ^6^Faculty of Health Sciences, Department of Physiatry and Nursing, University of Zaragoza, Zaragoza, Spain; ^7^HEME (Health, Economy, Motricity and Education) Research Group, University of Extremadura, Cáceres 10003, Spain; ^8^GENUD Toledo Research Group, Universidad de Castilla-La Mancha, Toledo, Spain; ^9^CIBER of Frailty and Healthy Aging (CIBERFES), Madrid, Spain; ^10^ImFine Research Group, Facultad de Ciencias de la Actividad Física y del Deporte-INEF, Universidad Politécnica de Madrid, Madrid, Spain; ^11^Centro de Investigación Biomédica en Red de Fisiopatología de la Obesidad y Nutrición (CIBERObn), Spain

## Abstract

The main objective of this study was to device-assess the levels of physical activity and sedentary behaviour patterns of older adults during the situation prior to the COVID-19 pandemic, home confinement, and phase-0 of the deescalation. We also aimed to analyse the effectiveness of an unsupervised home-based exercise routine to counteract the potential increase in sedentary behaviour during the periods within the pandemic. A total of 18 noninstitutionalized older adults(78.4 ± 6.0 y.), members of the Spanish cohort of the EXERNET-Elder 3.0 project, participated in the study. They were recommended to perform an exercise prescription based on resistance, balance, and aerobic exercises during the pandemic. Wrist triaxial accelerometers (ActiGraph GT9X) were used to assess the percentage of sedentary time, physical activity, sedentary bouts and breaks of sedentary time. An ANOVA for repeated measures was performed to analyse the differences between the three different periods. During home quarantine, older adults spent more time in sedentary behaviours (71.6 ± 5.3%) in comparison with either the situation prior to the pandemic (65.5 ± 6.7%) or the ending of isolation (67.7 ± 7.1%) (all *p* < 0.05). Moreover, participants performed less bouts of physical activity and with a shorter duration during home quarantine (both *p* < 0.05). Additionally, no differences in the physical activity behaviours were found between the situation prior to the pandemic and the phase-0 of deescalation. According to our results, the home confinement could negatively affect health due to increased sedentary lifestyle and the reduction of physical activity. Therefore, our unsupervised exercise program does not seem to be a completely effective strategy at least in this period.

## 1. Introduction

The coronavirus disease-19 (COVID-19) pandemic is an unprecedented health crisis that has forced millions of people to live in isolation as one of the main government actions to reduce the risk of spread. In Spain, the strict lockdown lasted 98 days (March-May 2020). This period began with home confinement (HC) and progressed through progressive deescalation phases with softer restrictions until the so-called “new normality.” During the HC, the Spanish population was only allowed to leave their homes to go to medical appointments or to do essential shopping. Afterwards, one of the first measures adopted during the initial phase of the deescalation (phase-0) was the possibility of doing physical activity outdoors in specific schedules according to age. Specifically, people over the age of 65 could go out on the streets from 10 to 12 a.m.

Unavoidably, these restrictions are repeating again in the present COVID-19 world situation and have modified the routine activities by increasing sedentary time [1, 2], so the pandemic may carry considerable risks to health and well-being [3–8]. Furthermore, COVID-19 spread is especially important in people at increased risk for severe illness like older adults.

Sedentary behaviour (SB), defined as any waking behaviour characterized by an energy expenditure < 1.5 METs (B. E. [9]), has been considered by some research as a new risk factor among the older adults, even regardless of physical activity (PA) levels [10]. SB is related to an increased risk of cardiovascular disease, diabetes, mental health problems, and some types of cancer, as well as premature all-cause, cancer, and cardiovascular disease mortality [11–14]. On the other hand, an increase in PA levels has been proposed as a relevant strategy to achieve successful aging [15–17] due to its positive health and fitness benefits [18, 19]. Nevertheless, older adults are characterized by a very sedentary lifestyle (only over 20% follow the PA recommendations) [20]. The most widely used tool to measure PA and SB is the self-reporting questionnaires [21, 22], although it is known that older adults tend to underestimate the time spent in sedentary activity and overestimate PA levels when subjective measurements are compared with accelerometers [23, 24].

Given the negative health consequences of reducing PA and increasing SB, from the beginning of the COVID-19 outbreak, some authors proposed continuing PA at home to stay healthy and maintain immune system function in the current precarious environment [3, 25]. Such forms of exercise may include strengthening exercises, balance activities, walking at home, stretching, or a combination of all or some of them [3]. Considering all the above, a home-based exercise routine prescription based on safe, simple, and easily implementable exercises in reduced spaces, could be an effective strategy to prevent or attenuate the effects of the potential increase in inactivity following the enforced lockdown in this population.

Up to now, little is known about the impact of home confinement caused by the COVID-19 pandemic on PA and SB of older adults. Besides, the few studies that exist on this topic have been carried out with subjective methods as questionnaires or surveys [2, 26]. Therefore, the main aim of this study was to objectively evaluate the differences in PA, SB, and break of sedentary time (BST) between the situation prior to COVID-19 pandemic, HC, and the phase-0 of the deescalation, analysing the effectiveness of an unsupervised home-based exercise routine to combat the potential increase in SB during the periods within the pandemic.

## 2. Methods

### 2.1. Study Design and Participants

Participants of the study were recruited from a Spanish cohort of the EXERNET-Elder 3.0 project, which is in line with the EU strategy “Health 2020,” focused on promoting active aging throughout life. The inclusion criteria to participate in this project were as follows: be older than 65 years, and not being dependent, or institutionalized. The exclusion criterion was suffering from cancer or dementia, because it was understood that dementia is incompatible with a fully independent life, and cancer is a nonchronic pathology with a high prevalence subject to treatments and changes that affect body composition and lifestyles. Participants of the project were initially allocated into two groups and were carrying out a supervised exercise interventions of 6-month duration from January 2020. The multicomponent training group performed three sessions per week of 1-hour duration, to improve endurance, strength, flexibility, balance, coordination, and functional capacity. On the other hand, the aerobic training group carried out once a week an endurance training session of 1-hour. All the sessions were divided into 10-15 min of warm-up (with joint mobility and cardiorespiratory exercises), 35-40 min of main exercises, and a 10 min cool down consisting of flexibility exercises. Nevertheless, the interventions were suddenly interrupted in March 2020 due to the COVID-19 pandemic. Afterwards, those members of the project who were habitually exercisers (participants of multicomponent training group) were invited to participate in this nonrandomized and noncontrolled trial. The invitations were done by phone on April 25 and 26, 2020, and there were fourteen participation refusal, twelve because they did not want to participate in the study and two because they were outside of the city. Thus, the sample of the study was a convenience subsample.

### 2.2. Functional Capacity, Body Composition, and Other Health-Related Evaluations

Functional capacity was assessed by Short Physical Performance Battery (SPPB) [27–29]. Besides, body mass index (BMI) was calculated by dividing weight (kg) by squared height (m^2^). Height was assessed by a portable stadiometer with 2.10 m maximum capacity and 1 mm error margin (Seca, Hamburgo, Germany), whereas a body composition analyser based on Bio-Electrical Impedance Analysis with 200 kg maximum capacity and 50 g error margin (TANITA BC-418MA, Tanita Corp., Tokyo, Japan) was used to measure the body weight (kg) and to estimate the percentage of body fat. Other variables included in this report to describe the sample status were as follows: Instrumental Activities of Daily Living Scale [30], Barthel Index [31], Mini Nutritional Assessment [32], and Mini Mental State [33].

### 2.3. Physical Activity and Sedentary Behaviour Assessment

PA and SB were evaluated with an ActiGraph GT9X triaxial accelerometer (ActiGraph GT9X Link; ActiGraph, 49 E. Chase St. Pensacola, FL 32502). Accelerometer output is an activity count, which is the weighted sum of the number of accelerations measured over a time period. The intensity of activity is assessed from the weighted sums, which are proportional to the magnitude of measured acceleration [34].

Participants wore the devices on their nondominant wrist, also when sleeping and removing them only for water-based activities (bathing or showering). Accelerometer data were collected at 60 Hz and were later aggregated into 60-second epochs. Nonwear time was defined by an interval of at least 60 consecutive minutes of zero activity intensity counts, with an allowance for 1-2 minutes of counts between 0 and 100 [35]. A SB was considered when the monitor registered <1853 counts per minute [36], while data ≥ 1853 counts were included as PA. In a further analysis of SB, three different aspects were evaluated: the percentage of wear time spent in SB, bouts of SB which were defined as periods of at least 10 consecutive minutes of SB and BST, which were considered as any interruption of SB independently of its duration. Regarding bouts of PA, a minimum block of 30 consecutive minutes was required, as the minimum estimated duration of the home-based exercise session.

Participants were familiar with the accelerometers as they had previously used them in previous phases of the EXERNET-Elder 3.0 project. In this study, they wore the devices in three different periods: (1) usual lifestyle (UL) prior to COVID-19 pandemic (December 2019): during this period, the participants were asked to maintain their daily routines unchanged for seven consecutive days; (2) the last two days of HC (April 30 and May 1, 2020): throughout this period (March 15-May 1, 2020), elderly participants stayed at home, and they were only allowed to leave them for medical appointments or shopping for basic needs; (3) first two days of phase-0 (2-3 May 2020): at this point, the confinement was lifted for a period of three hours per day, although older adults could go out only one hour per day. Additionally, for the periods within the pandemic (periods 2 and 3), participants were advised to incorporate a home-based exercise prescription into their daily routine. The accelerometers and instructions were given to the participants personally for the UL assessment and sent by express postal delivery services to their homes for the evaluations of the periods within the pandemic.

The data were analysed with the ActiLife software (ActiLife v6.13.3, ActiGraph Corp., Pensacola, Florida). Sleeping periods, nonwear time, and any day with less than 10 hours of wear time were removed from the analysis. Additionally, to be included in the sample, participants were required to have at least one valid day in all evaluation periods [36].

### 2.4. Implementation and Adherence of Unsupervised Exercise Training

With the main aim to engage older adults to perform the recommended levels of PA even at home and limit the sitting time in the COVID-19 pandemic [25], participants received during HC (by express postal service together with the accelerometer) a voluntary home-based exercise routine prescription. It was recommended to be performed five days per week from the last week of home confinement until the end of deescalation process.

The prescription was based on a specific literature review [37–39], and it was developed with the aim to be able to be performed at home, without sport equipment and through simple exercises with which the participants were familiarized previously during the intervention phase of the project in which they were involved. It consisted of seven resistance exercises, four for lower (chair squat, hip abduction and adduction, and calf raise) and three for upper limbs (push-ups on the wall, biceps curl, and shoulder abduction) carried out in two sets of 12-15 repetitions using the resistance of body weight and lifting home-available light free weights as liquid bottles; three balance exercises (semitandem or tandem position, single foot stand, and dynamic balance heel-toe) executed twice along 20 seconds, two times each one; and 20-30 minutes walking performed in one or several sets of 10 minutes minimum. The strength and balance exercises were performed twice each one by circuit-type protocol, being the resting time between sets and exercises of 45 seconds.

Furthermore, in order to analyse the adherence of the participants to the unsupervised home-based exercise routine, they were asked to register if they performed the training session during the days in which they had the accelerometers.

### 2.5. Statistical Analysis

Normality of the sampling distribution was proved using the Shapiro-Wilk test, and those variables which did not follow a normal distribution were transformed using the square root and the reverse function. Statistical significance was set at level *p* < 0.05 in all tests. Descriptive statistics (mean ± standard deviation (SD)) were calculated for all analysed variables, and an ANOVA for repeated measures was done to examine the differences in SB, PA, and sedentary breaks between the three different periods. For those variables transformed to normalized, the original mean and SD values were reported. Finally, a *t*-test for related samples was done to compare the percentage of time spent in PA between the schedule in which older adults could leave their homes, and the rest of time when they stayed at home during phase-0.

All the analyses were performed using the Statistical Package for Social Sciences software (SPSS, v. 25.0 for WINDOWS; SPSS Inc., Chicago, IL, USA), and values of *p* < 0.05 were considered statistically significant.

The general protocol of the EXERNET-Elder 3.0 study has been approved by the Ethics Committee of Clinical Research from the Alcorcón Foundation University Hospital (16/50); additionally, the Research Ethics Committee of the Autonomous Community of Aragon approved the specific study during the home quarantine (n° 10/2020). The whole study followed the ethical guidelines of the Declaration of Helsinki 1964, revised in Fortaleza (2013). Participants received oral and written information detailing the purpose, procedures, benefits, and risks that might result from their participation. All who voluntarily agreed to participate signed informed consent prior to first evaluation and in advance to the home quarantine evaluation.

## 3. Results

Data from six participants could not be included in the analysis due to delay in delivery of accelerometers by the express postal delivery service, so finally, data from 18 older adults were included. Descriptive characteristics of the participants at baseline are shown in Table 1.

### 3.1. Device-Assessed Data across Time Points and Effectiveness of Home-Based Exercise Routine

Participants wore the accelerometers for an average of 6.9 ± 0.2 days (1046.2 ± 86.1 minutes per day) in UL, 1.7 ± 0.5 days (936.1 ± 146.8 minutes per day) in HC, and 2.0 ± 0.0 days (1066.4 ± 130.0 minutes per day) in phase-0.  Table 2 shows the results of the analysed variables. The effectiveness of the unsupervised home-based exercise prescription was evaluated through the variables related to the device data (SB and PA variables).

During UL, participants had fewer sedentary bouts than in HC and phase-0 (*p* < 0.05), although there was no difference on its average duration among periods. Furthermore, in HC, older adults spent a higher percentage of wear time in SB compared with UL and phase-0 (*p* < 0.05), although no differences were found in BST. Regarding PA, during HC, elders performed fewer bouts and their average duration was shorter than in UL (*p* < 0.05).

### 3.2. Adherence of Unsupervised Home-Based Exercise Program

Regarding participants' compliance of the unsupervised home-based exercise routine during HC, 22.2% of older adults carried it out both days, 39.9% only one and the other 38.9% did not perform it. On the other hand, in phase-0 period, only 11.1% performed the training prescription two days, while 50% only one and the other 38.9% did not carry out any training session.

### 3.3. Physical Activity during Period Restrictions Was Lifted in Phase-0 of Deescalation

Additionally, during the two days of phase-0, 44.4% of older adults went out of home both days during the schedule in which restrictions were lifted, while 27.8% of the participants went out only one day and the other 27.8% did not go out any day. Furthermore, the average time spent in PA during the periods in which older adults were able to leave their homes was significantly higher than when restrictions were activated during phase-0 (49.2 ± 14.2% vs. 29.0 ± 7.5%; *p* < 0.001).

## 4. Discussion

The main findings of this study are as follows: (i) despite unsupervised training, during home confinement, elders spent more sedentary time than in usual lifestyle and during the phase-0 of deescalation; (ii) there were no differences in the breaks of sedentary time patterns between usual lifestyle and the periods within the pandemic; (iii) during home confinement, older adults performed fewer and shorter PA bouts than in the usual lifestyle despite unsupervised training.

### 4.1. Changes in Habits: Sedentary Behaviour, Physical Activity, and Breaks of Sedentary Time

To the best of our knowledge, no previous study has analysed through device-assessment the SB and PA patterns during the pandemic, and moreover, wrist accelerometers have shown to increase adherence to wear protocols [40]. The time spent in SB by our participants ranged between 66% of waking time in UL and 72% in HC. Long periods of SB during UL ranging between 65 and 80% of waking time (an average of 9.4 h/day) have also been reported in this population by a previous systematic review that analysed data obtained by accelerometry [24]. It is known that prolonged SB increases the risk of chronic diseases and mortality [10], so according to our results, the increase of SB during HC could adversely affect health [10].

In accordance with a recent study which aimed at analysing changes in activity and daily routine among Chinese citizens during home confinement, the prevalence of insufficient PA in this population rose over 4-fold during the epidemic quarantine (57.5%), compared with the nonepidemic period (14.1%) [7]. Moreover, the authors concluded that participants' screen time increased as PA level declined in the HC. Some of these conclusions could be extended internationally, since another recent research that obtained its data through an online international survey (of people aged over 18, including group over 55) concluded that the COVID-19 home confinement had a negative effect on all PA intensity levels and, additionally, increased daily sitting time in a 29% [1]. In our study, the increase of SB during the pandemic was much lower (6.1% in HC; 2.2% in phase-0) in comparison with UL. This fact may be partially explained because, with aging, physical activity decreases [35] and the previous study does not include specific data neither in older adults, who are characterized by a very sedentary lifestyle [41, 42], with only a small part of them following the PA recommendations of the World Health Organization [43]. Different factors may work together leading to an increase of SB during the periods within the pandemic. Regarding this issue, a current review of mental health problems in COVID-19 pandemic suggests that people affected by the pandemic may have a high burden of mental health problems, including depression, anxiety disorders, and stress [44]. In this way, a recent study with Spanish frail older adult community dwellers reported that among others, depressive symptoms and living alone decreased the odds of maintaining sufficient PA. Likewise, low PA levels are associated with increased prevalence of anxiety [45]. Consequently, all the above reinforces the importance of the promotion-specific plans, such as home-based exercise routines, or even a combination of both formal and informal activities to maintain PA levels in older people during lockdowns [25].

Regarding BST, a similar result (5.3 breaks/hour) was obtained in a previous study with frail older adults in UL [34] and Dos Santos et al. [46] obtained a greater amount (174 breaks/day) in nonfrail older adults, although the accelerometers were located on the hip in both studies. Previous research has shown that greater number of breaks in sitting behaviours is associated with better physical function [47] and longer SB bouts are detrimental to overall health [42]. Nevertheless, a recent study concluded that breaking-up sedentary time more often reduces frailty only in those older adults who are inactive [48]. Considering that older adults spent a longer time in SB during HC in our study, the absence of differences between periods in BST (n/hour) could suggest that along SB in HC, participants performed fewer BST than in the other two periods.

### 4.2. Effectiveness and Adherence of Unsupervised Home-Based Exercise Program

With regard to unsupervised training program, although it was not enough to avoid the differences with UL, it cannot be affirmed that it has not helped to partially avoid a greater sedentary time or mitigate a possible decline of fitness levels, having a positive effect on health. Moreover, two aspects should be taken into consideration. Firstly, the difficulty of walking at home, especially for older people, and secondly, most of strength and balance exercises had not increased the PA too much. The low compliance of the exercise program could be explained because unsupervised training was not motivating enough to create the necessary adherence in older adults, even though they were performing a supervised exercise program previously. This undoubtedly affected the results and could be the reason why the elderly did not maintain the SB patterns of UL during HC. On the other hand, the decrease of SB and the greater number and longer duration of PA bouts in phase-0 compared to HC could be associated with the possibility of going out of home for an hour per day, rather than the fact of doing more training sessions, given the low compliance of the exercise program and the increase of PA time during the schedule in which restrictions were lifted. In this regard, the absence of differences between phase-0 and UL in all variables except in the number of sedentary bouts per hour should be taken with caution, because the data of phase-0 was taken in the first two days when the restrictions were partially lifted. Consequently, older adults could increase their PA due to their desire to go out for a walk, although this behaviour might not be maintained over time.

### 4.3. Future Perspectives

Taking into account the adverse effects of sedentary lifestyle and the negative consequences of COVID-19 in PA and SB patterns of older adults, our findings can be used as a starting point to develop strategies to promote PA and reduce SB among older adults during similar situations of enforced lockdowns. Further research is needed to analyse the effectiveness of implementing motivational strategies or semisupervision in terms of improving adherence. Regarding fitness impact, although little is known about the dose-response relationship, previous systematic review has shown the greater benefits of supervised with respect to unsupervised strength and/or balance programs, being particularly prominent when compared with completely unsupervised [49]. The use of eHealth and exercise videos, which focuses on encouraging and delivering PA interventions through the Internet, mobile technologies, and television [50], is other viable avenues for maintaining physical function and mental health during this critical period [3]. A recent study has demonstrated, through Google Relative Search Rate, that the community interest in exercise surged following the lockdown [4]. Nevertheless, older adults tend to lag behind in their adoption of technology. In a study of 2014, very few seniors (18%) were smartphone users, although 59% reported using Internet [51]. However, the study was done in EEUU, so in other countries (especially the less developed), the results could be different. Furthermore, data showed a significant year-to-year increase in the internet adoption, so it is probably that senior users are higher nowadays. Nevertheless, and according to this information, a good approach would be to get older adults to be able to use new technologies efficiently.

### 4.4. Strengths and Limitations of This Study

The current research is highly topical and relevant, since we have not yet defeated the pandemic. Quarantines and confinements are repeated in Spain and different parts of the world. To our knowledge, this is the first study that device-assessed the impact of home confinement caused by COVID-19 in PA and SB patterns of older adults, while undergoing an unsupervised exercise intervention.

Nevertheless, some limitations of our study should be mentioned. Firstly, the small sample size was conditioned by the number of participants of the multicomponent training group of the EXERNET-Elder intervention that accepted to participate during the specific period of home confinement. Hence, this limits the generalisation of the results and our findings should be interpreted considering the absence of a control group for ethical reasons [52]. Secondly, although the attendance was registered, the level of compliance of the home-based exercise prescription could not be recorded because it was an unsupervised training. And thirdly, participants wore the accelerometer fewer days during the periods evaluated within the pandemic. This was because when the study was designed, the initial aim was to evaluate the physical activity and sedentary behaviour during usual lifestyle and home quarantine. Nevertheless, the government restrictions changed once the accelerometers were sent to the participants and the last two days of the programmed evaluation coincided with the first two days of phase-0; therefore, this limited the possibility to measure for more days but gave us the opportunity to analyse the possible differences between them, given the different restrictions between these two phases. Nevertheless, it is probable that, due to the restrictions imposed, daily routines were similar within the same periods and there would be no differences between weekdays and weekends, as previous studies have shown in this population in a usual life situation [53]. Moreover, specific cut points for light, moderate, and vigorous PA have not yet been defined for this population and body location for ActiGraph accelerometers, so given the importance of a deeper understanding of the PA patterns in healthy aging, future research effort should be made in this direction. Additionally, it should be highlighted that COVID-19 outbreak is leading to additional concerns and mental health problems which influence in daily behaviours [54] and their impact was not controlled in this study.

## 5. Conclusions

According to our results, the main conclusions are as follows: (1) home confinement increased the sedentary lifestyle in addition to causing a reduction of PA, which may lead to negative health effects in this population; (2) it seems that our unsupervised home-based exercise routine is not a completely effective strategy to maintain usual sedentary and PA patterns during the lockdown.

Confinements that are currently happening in the world show that the COVID-19 pandemic has not been defeated yet. Additionally, health recommendations to limit exposure and stay at home for as long as possible may increase the probability of semi-self-confinement in older people. Therefore, the situation stresses the need for more research looking for a health promotion guideline showing effective strategies in terms of maintaining daily PA routines, especially in older adults, the most exposed group to excessive SB.

## Figures and Tables

**Table 1:** Participant characteristics at baseline.

Age (years)	78.4 ± 5.4
Sex	
Male	7 (38.9)
Female	11 (61.1)
SPPB score	10.7 ± 2.7
Prefrail	4 (22.2)
Robust	14 (77.8)
BMI	29.7 ± 4.9
%BF	36.9 ± 7.0
IADL score	7.7 ± 1.0
Barthel Index score	99.4 ± 2.4
MNA score	26.6 ± 1.7
Minimental score	27.5 ± 2.2

Note: number of participants of the sample (*n*) and % per group for categorical variables; mean and standard deviation (S.D.) for continuous variables. SPPB: Short Physical Performance Battery; BMI: body mass index; %BF: body fat mass percentage; IADL: instrumental activities of daily living; MNA: Mini Nutritional Assessment.

**Table 2:** Participants' SB and PA patterns in the different evaluation periods.

	Usual lifestyle	Home confinement	Phase-0 of deescalation
SB (% total wear time)	65.5 ± 6.7	71.6 ± 5.3^ac^	67.7 ± 7.1
BST (number/h)	5.0 ± 0.5	4.6 ± 0.8	4.6 ± 0.8
10 min SB bouts (number/h)	0.9 ± 0.1^bc^	1.1 ± 0.2	1.1 ± 0.2
AVG time of 10 min SB bouts (min/bout)	30.4 ± 5.5	32.0 ± 8.9	29.4 ± 7.3
30 min PA bouts (number/day^∗^)	3.2 ± 1.7	2.2 ± 1.9^ac^	3.2 ± 2.1
AVG time of 30 min PA bouts (min/bout)	41.4 ± 5.2	31.7 ± 15.6^a^	42.1 ± 9.09

Note: SB: sedentary behaviour; BST: break sedentary time; AVG: average; PA: physical activity; ^∗^adjusted by 24 valid hours; ^a^significant difference with UL; ^b^significant difference with home confinement; ^c^significant difference with phase-0 of deescalation.

## Data Availability

The data used to support the findings of this study are available from the corresponding author upon request.

## Abbreviations

COVID-19: Coronavirus disease-19
MET: Metabolic equivalent of a task
PA: Physical activity
SB: Sedentary behaviour
BST: Break of sedentary time
UL: Usual lifestyle
HC: Home confinement
Phase-0: Phase-0 of the deescalation
SD: Standard deviation.

## References

[1] Ammar A., Brach M., Trabelsi K. (2020). Effects of COVID-19 home confinement on eating behaviour and physical activity: results of the ECLB-COVID19 international online survey. *Nutrients*.

[2] Suzuki Y., Maeda N., Hirado D., Shirakawa T., Urabe Y. (2020). Physical activity changes and its risk factors among community-dwelling Japanese older adults during the COVID-19 epidemic: associations with subjective well-being and health-related quality of life. *International Journal of Environmental Research and Public Health*.

[3] Chen P., Mao L., Nassis G. P., Harmer P., Ainsworth B. E., Li F. (2020). Coronavirus disease (COVID-19): the need to maintain regular physical activity while taking precautions. *Journal of Sport and Health Science*.

[4] Ding D., Cruz B. D. P., Green M. A., Bauman A. E. (2020). Is the COVID-19 lockdown nudging people to be more active: a big data analysis. *British Journal of Sports Medicine*.

[5] Narici M., De Vito G., Franchi M. (2021). Impact of sedentarism due to the COVID-19 home confinement on neuromuscular, cardiovascular and metabolic health: physiological and pathophysiological implications and recommendations for physical and nutritional countermeasures. *European Journal of Sport Science*.

[6] Owen N., Sparling P. B., Healy G. N., Dunstan D. W., Matthews C. E. (2010). Sedentary behavior: emerging evidence for a new health risk. *Mayo Clinic Proceedings*.

[7] Qin F., Song Y., Nassis G. P. (2020). Physical activity, screen time, and emotional well-being during the 2019 novel coronavirus outbreak in China. *International Journal of Environmental Research and Public Health*.

[8] Wang C., Pan R., Wan X. (2020). Immediate psychological responses and associated factors during the initial stage of the 2019 coronavirus disease (COVID-19) epidemic among the general population in China. *International Journal of Environmental Research and Public Health*.

[9] Ainsworth B. E., Haskell W. L., Whitt M. C. (2000). Compendium of physical activities: an update of activity codes and MET intensities. *Med. Sci. Sports Exerc*.

[10] Ekelund U., Tarp J., Steene-Johannessen J. (2019). Dose-response associations between accelerometry measured physical activity and sedentary time and all cause mortality: systematic review and harmonised meta-analysis. *The BMJ*.

[11] Biswas A., Paul I. O., Faulkner G. E. (2015). Sedentary time and its association with risk for disease incidence, mortality, and hospitalization in adults a systematic review and meta-analysis. *Annals of Internal Medicine. American College of Physicians.*.

[12] Koster A., Caserotti P., Patel K. V. (2012). “Association of sedentary time with mortality independent of moderate to vigorous physical activity.” Edited by Jonatan R. Ruiz. *PLoS ONE*.

[13] Patterson R., McNamara E., Tainio M. (2018). Sedentary behaviour and risk of all-cause, cardiovascular and cancer mortality, and incident type 2 diabetes: a systematic review and dose response meta-analysis. *European Journal of Epidemiology. Springer Netherlands.*.

[14] Thorp A. A., Owen N., Neuhaus M., Dunstan D. W. (2011). Sedentary behaviors and subsequent health outcomes in adults: a systematic review of longitudinal studies, 1996-2011. *American Journal of Preventive Medicine.*.

[15] Gopinath B., Kifley A., Flood V. M., Mitchell P. (2018). Physical activity as a determinant of successful aging over ten years. *Scientific Reports*.

[16] Paterson D. H., Warburton D. E. R. (2010). Physical activity and functional limitations in older adults: a systematic review related to Canada’s physical activity guidelines. *International Journal of Behavioral Nutrition and Physical Activity*.

[17] Rowe J. W., Kahn R. L. (1997). Successful aging. *The Cerontologist*.

[18] Allen J., Morelli V. (2011). Aging and exercise. *Clinics in Geriatric Medicine*.

[19] Taylor D. (2014). Physical activity is medicine for older adults. *Postgraduate Medical Journal.*.

[20] Hallal P. C., Andersen L. B., Bull F. C., Guthold R., Haskell W., Ekelund U. (2012). Global physical activity levels: surveillance progress, pitfalls, and prospects. *The Lancet*.

[21] Ainsworth B., Cahalin L., Buman M., Ross R. (2015). The current state of physical activity assessment tools. *Progress in Cardiovascular Diseases*.

[22] Madden K. M., Ashe M. C., Chase J. M. (2015). Activity profile and energy expenditure among active older adults, British Columbia, 2011-2012. *Preventing Chronic Disease*.

[23] Grimm E. K., Swartz A. M., Hart T., Miller N. E., Strath S. J. (2012). Comparison of the IPAQ-short form and accelerometry predictions of physical activity in older adults. *Journal of Aging and Physical Activity*.

[24] Harvey J. A., Chastin S. F. M., Skelton D. A. (2013). Prevalence of sedentary behavior in older adults: a systematic review. *International Journal of Environmental Research and Public Health.*.

[25] Wedig I. J., Duelge T. A., Elmer S. J. (2021). Infographic. Stay physically active during COVID-19 with exercise as medicine. *Br J Sports Med*.

[26] Pérez L. M., Castellano-Tejedor C., Cesari M. (2021). Depressive symptoms, fatigue and social relationships influenced physical activity in frail older community-dwellers during the Spanish lockdown due to the Covid-19 pandemic. *International Journal of Environmental Research and Public Health*.

[27] Guralnik J. M., Simonsick E. M., Ferrucci L. (1994). A short physical performance battery assessing lower extremity function: association with self-reported disability and prediction of mortality and nursing home admission. *Journals of Gerontology*.

[28] Izquierdo M. (2019). Multicomponent physical exercise program: Vivifrail. *Nutricion Hospitalaria*.

[29] Treacy D., Hassett L. (2018). The short physical performance battery. *Journal of Physiotherapy*.

[30] Lawton M. P., Brody E. M. (1969). Assessment of older people: self-maintaining and instrumental activities of daily living. *Gerontologist*.

[31] Mahoney F. I., Barthel D. W. (1965). Functional evaluation: the Barthel index. *Maryland State Medical Journal*.

[32] Guigoz Y., Vellas B., Garry P. J. (1997). Mini nutritional assessment : a practical assessment tool for grading the nutritional state of elderly patients. *Facts, Research and Intervention in Geriatrics*.

[33] Folstein M. F., Folstein S. E., McHugh P. R. (1975). "Mini-mental state": a practical method for grading the cognitive state of patients for the clinician. *Journal of Psychiatric Research*.

[34] Pozo-Cruz B., Mañas A., Martín-García M. (2017). Frailty is associated with objectively assessed sedentary behaviour patterns in older adults: evidence from the Toledo study for healthy aging (TSHA). *PLoS One*.

[35] Troiano R. P., Berrigan D., Dodd K. W., Mâsse L. C., Tilert T., Mcdowell M. (2008). Physical activity in the United States measured by accelerometer. *Medicine and Science in Sports and Exercise*.

[36] Koster A., Shiroma E. J., Caserotti P. (2016). Comparison of sedentary estimates between activPAL and hip- and wrist-worn ActiGraph. *Medicine and Science in Sports and Exercise*.

[37] Chodzko-Zajko W. J., Proctor D. N., Fiatarone Singh M. A. (2009). Exercise and physical activity for older adults. *Medicine and Science in Sports and Exercise.*.

[38] Fragala M. S., Cadore E. L., Dorgo S. (2019). Resistance training for older adults: position statement from the National Strength and Conditioning Association. *Journal of Strength and Conditioning Research*.

[39] Lopez P., Pinto R. S., Radaelli R. (2018). Benefits of resistance training in physically frail elderly: a systematic review. *Aging Clinical and Experimental Research*.

[40] Troiano R. P., McClain J. J., Brychta R. J., Chen K. Y. (2014). Evolution of accelerometer methods for physical activity research. *British Journal of Sports Medicine*.

[41] Colley R. C., Garriguet D., Janssen I., Craig C. L., Clarke J., Tremblay M. S. (2011). Physical activity of Canadian children and youth: accelerometer results from the 2007 to 2009 Canadian Health Measures Survey. *Health Reports*.

[42] Sardinha L. B., Santos D. A., Silva A. M., Baptista F., Owen N. (2015). Breaking-up sedentary time is associated with physical function in older adults. *Journals of Gerontology-Series A Biological Sciences and Medical Sciences*.

[43] World Health Organization (2002). *United Nations Department of Economics and Social Affairs: Population Division*.

[44] Hossain M. M., Tasnim S., Sultana A. (2020). Epidemiology of mental health problems in COVID-19: a review. *F1000Research*.

[45] Kim S. Y., Jeon S. W., Lee M. Y. (2020). The association between physical activity and anxiety symptoms for general adult populations: an analysis of the dose-response relationship. *Psychiatry Investigation*.

[46] Santos C. E. S. D., Manta S. W., Maximiano G. P. (2018). Accelerometer-measured physical activity and sedentary behavior: a cross-sectional study of Brazilian older adults. *Journal of Physical Activity and Health*.

[47] Blair C. K., Morey M. C., Desmond R. A. (2014). Light-intensity activity attenuates functional decline in older cancer survivors. *Medicine and Science in Sports and Exercise*.

[48] Mañas A., del Pozo-Cruz B., Rodríguez-Gómez I. (2021). Breaking sedentary time predicts future frailty in inactive older adults: a cross-lagged panel model. *The Journals of Gerontology: Series A*.

[49] Lacroix A., Hortobágyi T., Beurskens R., Granacher U. (2017). Effects of supervised vs. unsupervised training programs on balance and muscle strength in older adults: a systematic review and meta-analysis. *Sports Medicine. Springer International Publishing*.

[50] Tate D. F., Lyons E. J., Valle C. G. (2015). High-tech tools for exercise motivation: use and role of technologies such as the internet, mobile applications, social media, and video games. *Diabetes Spectrum.*.

[51] Smith A. (2014). Usage and Adoption|Pew Research Center. https://www.pewresearch.org/internet/2014/04/03/usage-and-adoption/.

[52] Izquierdo M., Rodriguez-Mañas L., Casas-Herrero A., Martinez-Velilla N., Cadore E. L., Sinclair A. J. (2016). Is it ethical not to prescribe physical activity for the elderly frail?. *Journal of the American Medical Directors Association*.

[53] Schlaff R. A., Baruth M., Boggs A., Hutto B. (2017). Patterns of sedentary behavior in older adults. *American Journal of Health Behavior*.

[54] Torales J., O’Higgins M., Castaldelli-Maia J. M., Ventriglio A. (2020). The outbreak of COVID-19 coronavirus and its impact on global mental health. *International Journal of Social Psychiatry. SAGE Publications Ltd.*.

